# Greater trochanteric osteotomy and subtrochanteric osteotomy in primary/revision total hip arthroplasty

**DOI:** 10.3389/fsurg.2023.1103689

**Published:** 2023-02-09

**Authors:** Yuqi Pan, Yunsu Chen

**Affiliations:** Department of Joint Surgery, Shanghai Sixth People's Hospital Affiliated to Shanghai Jiao Tong University School of Medicine, Shanghai, China

**Keywords:** osteotomy of the femur, primary THA, revision THA, greater trochanteric osteotomy, subtrochanteric osteotomy

## Abstract

Osteotomy of the femur is necessary in some cases of primary/revision total hip arthroplasty (THA) procedure. There are two mainly used femur osteotomy methods in THA: greater trochanteric osteotomy and subtrochanteric osteotomy. Greater trochanteric osteotomy can improve hip exposure, provide greater stability against dislocation and favorably influence the abductor moment arm. Whether in the primary or revision THA, greater trochanteric osteotomy has its unique position. Subtrochanteric osteotomy adjusts the degree of femoral de-rotation and corrects the leg length. It is widely used in hip preservation and arthroplasty surgery. All osteotomy methods have specific indications, while nonunion is the commonest complication. In this paper, we analyze the greater trochanteric osteotomy and the subtrochanteric osteotomy in primary/revision THA and summarize the characteristics of different osteotomy methods.

## Introduction

Osteotomy of the femur is not routinely performed in total hip arthroplasty (THA) surgery. But in some complex cases, femur osteotomy is an effective method. Various femur osteotomy methods have been described, of which greater trochanteric osteotomy and subtrochanteric osteotomy were the two commonly used methods. Greater trochanter, an important anatomical landmark of the femur, is often used as a reference for femur osteotomy in THA. Greater trochanteric osteotomy and subtrochanteric osteotomy are created according to the different osteotomy sites relative to the greater trochanter. And few studies were focusing on the greater trochanteric osteotomy and subtrochanteric osteotomy.

Greater trochanteric osteotomy was once used regularly in THA, but now its application is limited to complex primary/revision cases. There are many types: standard greater trochanteric osteotomy, modified greater trochanteric osteotomy (v-shaped, oblique, horizontal, vertical), greater trochanteric slide osteotomy, and extended greater trochanteric osteotomy.

Subtrochanteric osteotomy is mainly used in developmental dysplasia of the hip (DDH) cases. The variations of the subtrochanteric osteotomy mainly include transverse subtrochanteric osteotomy, oblique subtrochanteric osteotomy, step-cut subtrochanteric osteotomy, and double-V subtrochanteric osteotomy.

Currently, criteria on whether to perform femoral osteotomy in THA have not been standardized, and the amount of bone osteotomy remains a question in THA under various complex situations. There are many femoral osteotomy methods whether in the primary THA or revision THA. Some were popular in the past but are rarely used now and some new methods are emerging. This review intends to summarize greater trochanteric osteotomy and subtrochanteric osteotomy methods in primary THA or revision THA and explores the advantages and disadvantages of each method.

## Greater trochanteric osteotomy

**Figure 1:**
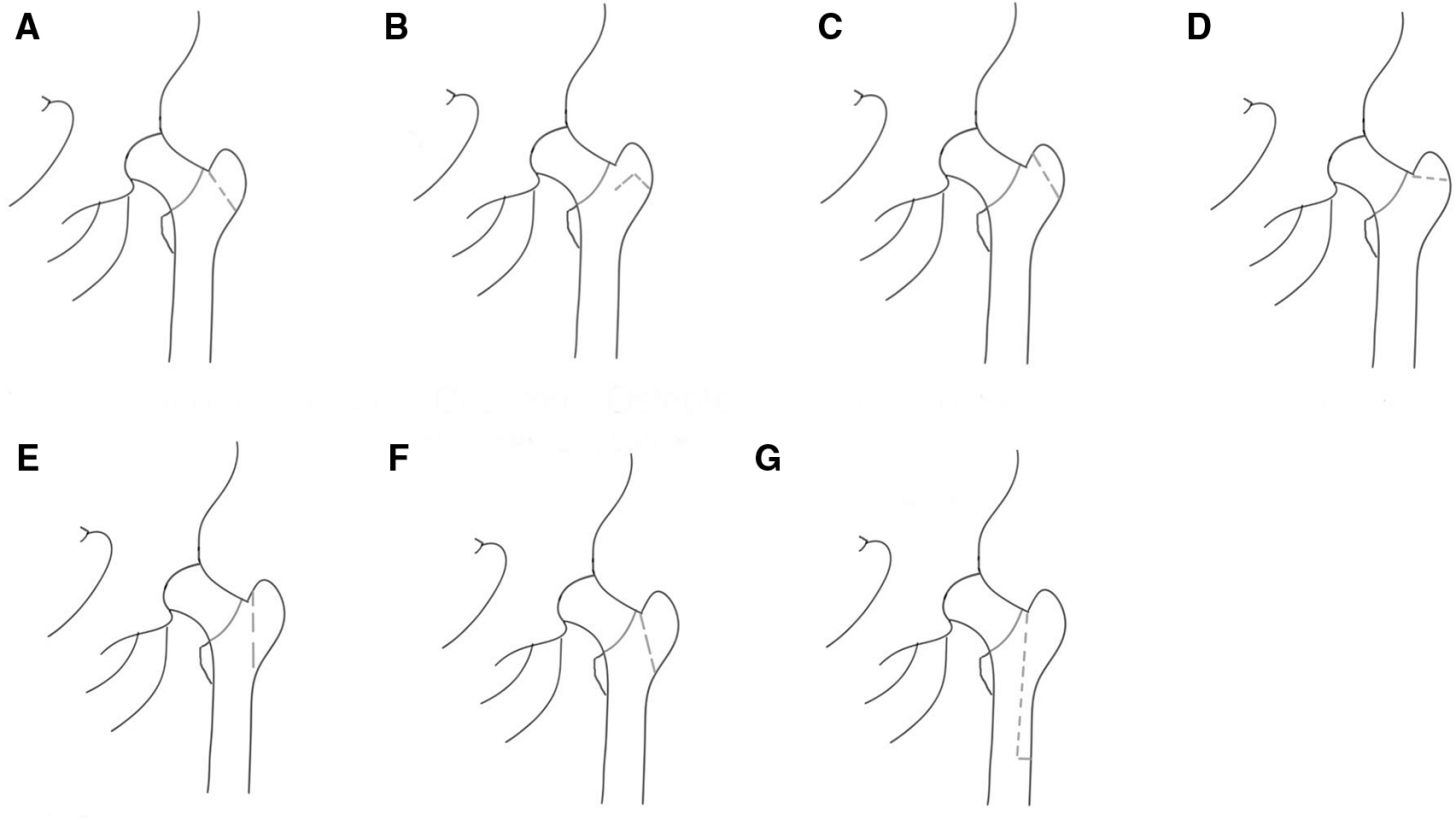
Greater trochanteric osteotomy methods. (**A**) Standard greater trochanteric osteotomy. (**B**) V-shaped greater trochanteric osteotomy. (**C**) Oblique greater trochanteric osteotomy. (**D**) Horizontal greater trochanteric osteotomy. (**E**) Vertical greater trochanteric osteotomy. (**F**) Greater trochanteric slide osteotomy. (**G**) Extended greater trochanteric osteotomy.

Except for the standard greater trochanteric osteotomy and modified greater trochanteric osteotomy (v-shaped, oblique, horizontal, vertical), greater trochanteric osteotomy also includes greater trochanteric slide osteotomy and extended greater trochanteric osteotomy, which expands its application in THA surgery. Generally, the greater trochanteric osteotomy increases the exposure of the acetabulum and the femur in complex primary and revision THA. Charnley believed this approach can help facilitate access to the hip joint, achieve correct alignment of the prosthetic components and permit the ability to favorably influence the abductor lever arm (1). Nonunion is the most important complication in the greater trochanteric osteotomy. The trochanter nonunion rate occurred from 1% to 38% (2) according to different studies. Other complications are hip pain; hip dislocation; heterotopic ossification; sciatic nerve injury; increment of blood loss and operative duration (3). Table 1 illustrates the indications and complications of each greater trochanteric osteotomy method. Figure 1 shows different osteotomy methods of the greater trochanter.

**Table 1:** Indications and complications of greater trochanteric osteotomy.

Types	Indications	Complication
Standard	Primary/Revision THA: Wider exposure of the hip Femur needs to be shortened Excessive laxity of the abductor muscle	Nonunion Trochanteric bursitis Heterotopic ossification
V-shaped	Primary THA: Modification of the standard osteotomy	Nonunion
Oblique	Primary THA: Patients at risk for posterior dislocation	Nonunion
Horizontal	Revision THA: Wider exposure of the hip Femur needs to be shortened Excessive laxity (compromised trochanteric bed)	Nonunion
Vertical	Revision THA: Previous surgery has moved the trochanter distally, to the lateral femoral cortex	Nonunion
Slide	Primary/Revision THA: Same as the standard osteotomy in Primary THA Acetabular revisions, protrusio acetabuli, and cemented femoral revisions in revision THA	Nonunion
Extended	Revision THA: Well-fixed stem requires revision; Wide exposure for the loose femoral components	Nonunion

### Standard greater trochanteric osteotomy

Greater trochanteric osteotomy was first introduced by Professor John Charnley (4) and it can be used in complex primary and revision THA cases. For example, when the femur must be shortened, surgeons should perform the osteotomy and advancement of the greater trochanter. Failure to advance the trochanter leaves the abductors lax, increasing the risk of limp and dislocation. Some complex primary or revision cases also require greater trochanteric osteotomy for enhanced exposure of the acetabulum and the proximal femur (5).

Standard greater trochanteric osteotomy is used in some infrequent primary THA that requires wider exposure. For instance, severe DDH or situations when the abductors' unacceptable laxity needs to be corrected (5). Another indication for the standard greater trochanteric osteotomy is the need for extensive acetabular exposure in complex acetabular revision such as implantation of an anti-protrusio cage with a large flange on the ilium (6).

Surgical methods include releasing the proximal portion of the vastus lateralis muscle origin and exposing the vastus tubercle; making the osteotomy cut transverse to sulcus between the lateral portion of the origin of the vastus intermedius muscle and the insertions of the gluteus medius and minimus; retracting the trochanteric proximally and releasing the external rotators; reattaching the trochanteric. The commonest complication is nonunion, and about 15%–20% occurred in revision cases according to the studies (7). Other reported complications included trochanteric bursitis and heterotopic ossification (5, 6). Since nonunion can be the underlying factor for hip pain, abductor muscle insufficiency, and hip instability, surgeons must apply firm fixation to reattach the great trochanter. It should provide compression to the osteotomy site and resist both vertical displacement and rotatory forces in the anteroposterior plane that is provided by the abductors with the hip in flexion (6).

### Modified standard greater trochanteric osteotomy

Modified standard greater trochanteric osteotomy can increase stability, reduce the incidence of nonunion and provide greater efficiency in some primary and revision THA cases. These modified forms include the chevron (V-shaped osteotomy), the oblique osteotomy, the horizontal osteotomy, and the vertical osteotomy.

Oblique and V-shaped osteotomy are mainly used in primary THA, while horizontal and vertical osteotomy are often used in revision THA (5).

The chevron osteotomy, with a “V-shaped” incision, provides inherent stability with resistance to rotation and anteroposterior displacement (6). The osteotomy is about 4–5 cm distal to the proximal tip of the great trochanter. The anterior and posterior osteotomy of the great trochanter are planned to be equal in size to form a biplanar surface that is convex laterally. The anterior trochanteric osteotomy is made at approximately 30° to the parasagittal plane while the posterior trochanteric osteotomy is made at 120°–130° to the first osteotomy (8).

The oblique osteotomy allows for more extensive exposure of the hip joint when performing direct lateral approach THA, providing bony healing to facilitate reattachment of the anterior abductor muscle. This osteotomy has the advantage of maintaining the continuity of the gluteus medius and lateral femoral muscles and the posterior portion of the trochanter (9). Anteriorly, the cut is parallel to the standard greater trochanteric osteotomy but slightly more craniad. The posterior portion of this osteotomy exits anteriorly to the intertrochanteric ridge. The entire insertions of the gluteus medius and minimus are on the osteotomized fragment, but the short external rotators and capsule remain uninterrupted (5).

In revision cases when normal greater trochanteric osteotomy does not preserve enough cancellous bone beds to reconnect the trochanter segments, horizontal greater trochanteric osteotomy enables additional exposure. The approach is similar to standard osteotomy. However, the osteotomy is performed at 70° to 90° to the femoral diaphysis, as close to the rotor as possible, so that the gluteus minimus and gluteus medius are left intact over the greater trochanter fragment. The trochanter fragment can be reattached to the lateral femoral cortex (6, 10).

Vertical greater trochanteric osteotomy is mainly used in revision THA cases in which the trochanter has already been advanced to the lateral femoral cortex during a previous trochanteric advancement. Wide exposure is necessary with a full release of the vastus intermedius and lateralis well down the femur, distal to the greater trochanter. The vertical greater trochanteric osteotomy runs parallel to the lateral cortex of the proximal femur, leaving enough cancellous bone (3 to 5 mm) on the lateral side of the cortex to reattach to the cancellous bone bed. The gluteus medius and gluteus minimus remain on the osteotomy fragment (5, 6).

### Greater trochanteric slide osteotomy

The abductor weakness is correlated with the amount of separation especially if it exceeds two centimeters (11). To avoid impaired abductor function associated with proximal displacement of non-united trochanteric segments, trochanteric slide osteotomy has been described. English et al. (12) first described the greater trochanteric slide osteotomy in primary THA, while Glassman et al. (13) supported its use in revision hip surgery in its modern form. High-dislocation DDH and revision THA in patients with abductor deficit are indications regarding the greater trochanteric slide osteotomy in THA surgery because the modified trochanteric slide osteotomy retains the posterior capsule, short external rotators, and acetabular exposure (14). Some scholars applied greater trochanteric slide osteotomy to stiff hip cases and recommended its use for wider exposure of the hip (15).

The principal surgical techniques include performing the short oblique osteotomy of the greater trochanter; anteriorly sliding the greater trochanter to facilitate exposure; implanting the femoral prosthesis; fixing the greater trochanter fragment (16). The greater trochanteric slide osteotomy maintains both the vastus lateralis and the glutei attachment, providing a compressive lateral force on the trochanter against the femur. This compression makes it stabler (2). Abductor function is preserved as it keeps the continuity of the abductor-lateralis myofascial sleeve (6). Professor Rocco P. Pitto put it that greater trochanteric slide osteotomy can be used in resurfacing hip arthroplasty and the procedure will not affect the proximal femur blood supply (17). For its use in high-dislocation DDH, Pan Y et al. included 52 patients (57 hips) with CroweIVDDH who underwent greater trochanteric slide osteotomy. In long-term follow-up, the authors found 51 hips (89.5%) achieved bony healing;4 hips (7.0%) had fibrous union and 2 hips (3.5%) had nonunion (16). Langlais et al. reported the results of 94 patients who had prosthetic loosening after primary THA and underwent the revision procedure using greater trochanteric slide osteotomy. They found 96% of patients achieved trochanteric union (7). B. Sonny Bal et al. studied 73 patients who underwent greater trochanter slide osteotomy at an average duration of 36 months after surgery and claimed that 67 (92%) of the greater trochanter had been reattached to the femur, and of the 6 trochanters that did not heal, 3 had fibrous unions *in situ* and the other 3 had lateral and anterior displacement relative to the original trochanter bed, with minimal displacement in the proximal direction (18). As for the fixation of the greater trochanter in greater trochanteric slide osteotomy, some professors thought the cable-plate and the cerclage wire both offered good fixation results but the cerclage wire had better clinical outcomes (2).

### Extended greater trochanteric osteotomy

Extended greater trochanteric osteotomy was popularized by Wagner and was later modified by Younger et al. (19). Indications for extended greater trochanteric osteotomy include removal of a well-fixed cemented or non-cemented femoral prosthesis in revision THA, remodeling of the varus proximal femoral, and the need for enhanced acetabular exposure. The length of the osteotomy needs to be planned preoperatively to provide adequate hip exposure and maintain at least 5 cm of the ischial diaphyseal cortex for prosthetic fixation. Typically, the osteotomy should be at least 10 cm long measured from the tip of the greater trochanter to allow fixation of the fragment to the medial femoral cortex. The length of the osteotomy in femoral prosthesis revision is typically 12 to 15 cm (6). It is hypothesized that a longer osteotomy range would improve the bone contact area and promote osteotomy healing. The anterolateral proximal femur is cut for one-third of its circumference, extended distally, and levered open on an anterolateral hinge of periosteum and muscle. This results in an intact muscle-osseous sleeve composed of the gluteus medius, greater trochanter, anterolateral femoral diaphysis, and vastus lateralis, as well as good exposure of the fixation surface and distal cement (19). It reduces the risk of greater trochanter displacement by keeping the soft tissue of the lateral femoral and anterolateral muscles attached to the osteotomy fragment, thus counteracting the coronal plane pull of the hip abductor muscles and generating a compressive force to prevent proximal displacement and further stabilizing the osteotomized greater trochanter (20).

A review study noted that the probability of bony healing of extended greater trochanteric osteotomy in revision THA due to various reasons (e.g., periprosthetic infection, aseptic loosening of the prosthesis, and periprosthetic fractures) averaged 95.2%, with a relatively low incidence of postoperative prosthetic subsidence, greater trochanteric displacement, and periprosthetic fractures (21). In another study of 612 patients who underwent hip revision surgery with the extended greater trochanteric osteotomy (median follow-up time of 5 years), the authors found the incidence of postoperative bone nonunion was only 2% and 98% of the osteotomy sites achieved bony healing within 6 months (22). Another study pointed out that even in the presence of the periprosthetic joint infection, extended greater trochanteric osteotomy remains a powerful tool for femoral component removal (23).

Although extended greater trochanteric osteotomy is mainly used in revision THA, its use in primary THA has also been reported (24). Luo et al. found good outcomes in 19 patients (23 hips) with high-dislocation DDH who underwent extended greater trochanteric osteotomy. They performed 8 cm to 12 cm extended greater trochanteric osteotomies to facilitate acetabular and femoral exposure and reported bony healing of the greater trochanter in all patients 1 year after the procedure (25).

## Subtrochanteric osteotomy

Subtrochanteric osteotomy is a common osteotomy for high-dislocation DDH. Femoral shortening can protect neurovascular structures as well as provide correction of the excessive femoral anteversion and lateral location of the abductor lever (26). It mainly consists of transverse subtrochanteric osteotomy, oblique subtrochanteric osteotomy, step-cut subtrochanteric osteotomy, and double-V subtrochanteric osteotomy.

**Figure 2:**
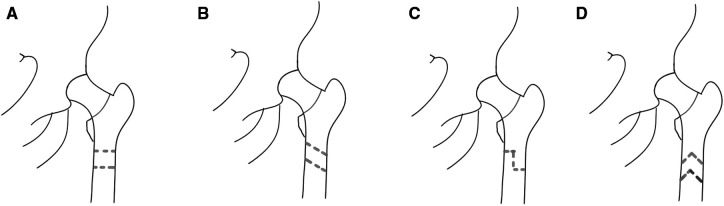
Subtrochanteric osteotomy methods. (**A**) Transverse subtrochanteric osteotomy. (**B**) Oblique subtrochanteric osteotomy. (**C**) Step-cut subtrochanteric osteotomy. (**D**) Double-V subtrochanteric osteotomy.

Previously reported incidences of nonunion in subtrochanteric osteotomies for high-dislocation DDH ranged between 2.8% and 7.1% (27). In the subtrochanteric osteotomy, it is important to fix the osteotomy site to provide a proper environment for bone healing and maintain the normal femoral anteversion angle, facilitating osseointegration of the femoral component. Fixation with plates and screws is one of the options (28). A study comparing the subtrochanteric osteotomy and trochanteric slide osteotomy has concluded that both methods have similar clinical outcomes in the treatment of Crowe III-IV DDH at midterm follow-up (uncemented THA). Bone healing rates were relatively higher in the greater trochanteric slide osteotomy, but the clinical results were not statistically significant in 5 years follow-up. The authors drew a conclusion that there is no significant advantage of subtrochanteric shortening osteotomy over trochanteric slide osteotomy, and both techniques can be used safely depending on surgeons' preference (29). Table 2 compares the advantages and disadvantages of transverse subtrochanteric osteotomy and other subtrochanteric osteotomies. Figure 2 shows different subtrochanteric osteotomy methods.

**Table 2:** Advantages and disadvantages of transverse subtrochanteric osteotomy and other subtrochanteric osteotomies.

Types	Advantages	Disadvantages
Transverse	Easy and simple Without any special instrument	May have higher nonunion rate
Oblique, step-cut and double-V	Provide greater rotational stability More bony surface contact	More complex Longer studying curve

### Transverse subtrochanteric osteotomy

It is the simplest subtrochanteric osteotomy technique, requiring only two transverse cuts of the femur below the lesser trochanter without the use of any special instruments (30) and also allowing for easier adjustment of the femoral rotation. Compared to other subtrochanteric osteotomy approaches, the transverse subtrochanteric osteotomy is simpler and easier to perform (31). A meta-analysis was performed to compare the influence of transverse and other subtrochanteric osteotomies in terms of patient complications and prosthesis survival rates, and no differences were found (32). Erhan Sukur et al. analyzed 56 patients (68 hips) with Crowe type IV DDH who were treated with uncemented subtrochanteric transverse osteotomy and found that most of the patients had good clinical outcomes at a mean follow-up of 12.9 years while 9(13.2%) patients had complications and 8(11.7%)patients had secondary revision surgery (27). Cagri Ors et al. retrospectively analyzed 91 Crowe IV hip dysplasia patients (127 hips) treated with transverse subtrochanteric osteotomy using Wagner-cone stem. The 10-year prosthetic survival rate was approximately 94.5%, and the femoral prosthesis survival rate was 96.9% through a mean follow-up of 8.4 years (33).

### Oblique, step-cut, and double-V subtrochanteric osteotomy

These osteotomies have inherent stability due to their three-dimensional geometry. Compared to transverse osteotomies, these osteotomies increase the bone-contacting surface, thus promoting the bone union. Subtrochanteric step-cut and double-V-shaped osteotomies are stabler in terms of rotation than transverse osteotomy. However, these osteotomies are technically complex and require some surgical experience and precise preoperative planning, especially when the femoral anteversion is corrected (28, 31).

The oblique osteotomy method is similar to transverse osteotomy, but the osteotomy surface is angled downward. A finite element analysis study of osteotomy angles showed that 45° is more appropriate, with minimal micromovement of the osteotomy surface among 30°, 45°, 60°, and 90° (34). Dogan Atlihan et.al compared the biomechanical stability of four different subtrochanteric osteotomy approaches and they found oblique osteotomy could provide a larger contact surface in the osteotomy to promote bone healing (35). Oblique subtrochanteric osteotomy can improve rotational stability compared to transverse subtrochanteric osteotomy. The procedure can be carried out easily by two parallel osteotomies. The oblique subtrochanteric osteotomy also has the benefit of allowing parallel osteotomies to gradually increase the amount of osteotomized bone (36).

Step-cut osteotomy also increases the bone contact area and provides rotational stability, but is more complex and requires a longer learning curve (37, 38). Compared to transverse subtrochanteric osteotomy, it has higher torsional strength (39). Subtrochanteric step-cut osteotomy can reduce circumferential contact between the endosteal cortex and the femoral stem, which provides an unstable femoral stem press-fit and results in an inversion deformity of the femoral stem (31). Step-cut subtrochanteric osteotomy consists of two transverse osteotomies that are half the width of the bone, with a longitudinal linear portion in a vertical position connecting two semi-transverse osteotomies. It is performed manually by the surgeon and requires more time and tissue exposure than transverse subtrochanteric osteotomy (39). Orhan Akıncı et al. followed 31 patients (35 hips) with high-dislocation DDH who underwent step-cut subtrochanteric osteotomy for a mean time of 9.2 years. The clinical and functional scores of patients were significantly improved after surgery. 9 patients had minor fractures in the intraoperative acetabular consultation, all of which were controlled. After 10 weeks, one patient developed local osteolysis and bone nonunion and recovered after 6 weeks with the treatment of plate and cable looping. Intraoperative fractures of the lateral femur occurred in 4 patients and they recovered well after cable looping. The survival rate of the prosthesis was 93% at the end of 5 years and 89% at the end of 9.2 years after surgery (38).

Becker and Gustilo described a “double V-shaped subtrochanteric rotational shortening osteotomy” that enabled greater torsional stability at the osteotomy site and fixation with a shorter femoral stem (40). It innovated the subtrochanteric transverse osteotomy by first completing a transverse osteotomy to shorten the femur, then adjusting the proximal and distal fragments to an appropriate anteversion, and finally reshaping the transverse osteotomy geometry into a double V-shape. Some professors suggested that the double-V osteotomy is simpler than the step-cut osteotomy and provides more rotational stability than the transverse osteotomy (41).

## Conclusion

Greater trochanteric osteotomy and subtrochanteric osteotomy are two main osteotomy methods in primary/revision THA. Trochanteric osteotomy includes standard greater trochanteric osteotomy, modified standard greater trochanteric osteotomy (v-shaped, oblique, horizontal, vertical), trochanteric slide osteotomy and extended greater trochanteric osteotomy. The major advantage of greater trochanteric osteotomy is the increasing exposure of the hip joint. The standard greater trochanteric osteotomy is not frequently used now and modified standard greater trochanteric osteotomy can increase the stability of the osteotomy. In modified greater trochanteric osteotomy, oblique osteotomy and V-shaped osteotomy are often used in primary THA, while horizontal and vertical osteotomies are mainly used in revision THA. Greater trochanteric slide osteotomy, which is used in both primary and revision THA, preserves the lateral femoral muscle and prevents proximal trochanter displacement. Extended greater trochanteric osteotomy is mainly used in revision THA, increasing the osteotomy surface and facilitating osteotomy healing.

Subtrochanteric osteotomies are widely used in primary THA, especially in high-dislocation DDH. It includes transverse, oblique, step-cut, and double-V subtrochanteric osteotomy. The transverse subtrochanteric osteotomy is simple to perform but is more prone to osseous nonunion. Other osteotomies increase the bone contact area and rotational stability, reducing the incidence of osseous nonunion. However, there are some drawbacks, such as surgical complexity and a longer learning curve.

Bone nonunion is a common issue in all osteotomies, and more solutions are needed. In primary/revision THA, it is critical to consider all osteotomy indications and select the appropriate osteotomy method for specific patient situations.

## Author contributions

All authors contributed to the article collection and analysis, manuscript writing and editing. All authors contributed to the article and approved the submitted version.

## Fundings

The review was funded by Shanghai Municipal Commission of Health And Family Planning Project: The role of digital gap balancer in optimizing soft tissue balance in total knee arthroplasty (numbers: 201840032).
